# Inoculation against Rinderpest

**Published:** 1898-08-27

**Authors:** 


### INOCULATION AGAINST RINDERPEST.

Professor Kohlstock, lecturing recently at Berlin, reported on the results of using Dr. Koch's lymph for the inoculation of cattle against rinderpest in South Africa. The experiments had been highly successful, and he stated that 30 per cent. of those treated belonging to natives, and more than double the number belonging to Europeans, were saved. He reported that after five months' use of the lymph, the carrying trade by means of oxen had been resumed, and that since the complete adoption of the process no fresh case of the pest had occurred.

